# Intrathoracic Desmoid Tumor

**DOI:** 10.5334/jbr-btr.834

**Published:** 2015-09-15

**Authors:** L. D’Hooghe, S. Dekeyzer, T. Dewaele, K. Gieraerts, K. Coenegrachts

**Affiliations:** 1 Department of Radiology and medical imaging, University Hospital Ghent, Ghent, Belgium; 2 Department of Radiology and medical imaging, AZ Sint-Jan Brugge-Oostende AV, Bruges, Belgium

A 47-year-old female was referred to the hospital because of persistent pain at the ventral right side of the chest for several weeks. Except for ankylosing spondylitis, her medical history was insignificant. The patient doesn’t smoke and there is no history of thoracic trauma or surgery. There were no alarm symptoms. Clinical examination revealed no abnormalities and routine hematologic and biochemical evaluation were normal.

Breast US was performed and incidentally a sharply delineated heterogeneous intra-thoracic soft tissue mass of 4.5 × 9 cm was observed arising from the right chest wall. Additional X-rays of the chest showed a large intra-thoracic right-sided mass with a broad pleural base, smoothly bordering the lung parenchyma (Fig. A). Contrast-enhanced CT of the chest confirmed the presence of a right-sided sharply delineated heterogeneous intra-thoracic mass with a broad pleural base (Fig. B). Bony alterations were present on the adjacent ribs. There were no enlarged lymph nodes, pleural fluid or intra-thoracic nodules. At MRI imaging, the mass had mixed signal intensity on T2WI and was iso-intense to skeletal muscle on axial 3D gradient-echo T1WI images. The lesion was irregularly marginated where it bordered the thoracic wall, suggesting growth in the intercostal spaces. Strong and heterogeneous enhancement occurred on T1WI after gadolinium administration (Fig. C). Finally PET-CT showed only mild, heterogeneous FDG-uptake in the mass, suggesting a benign lesion. Because malignancy could still not be ruled out, a surgical biopsy was performed. Pathologic examination showed a fibromatous spindle cell lesion without signs of high grade malignancy. No mitosis, nor necrosis nor pleomorphism were observed. Histological and immunohistochemical image was strongly suggestive for desmoid tumor with staining negative for keratin, CD34 and S100 and positive for beta-catenin and actin. These results were re-examined and confirmed in a second hospital. Surgery was recommended, but the patient rejected and chose radiotherapy. Follow-up imaging six months later showed slight volume loss of the tumor. The patient remains in further follow-up.

**Figures A–C:**
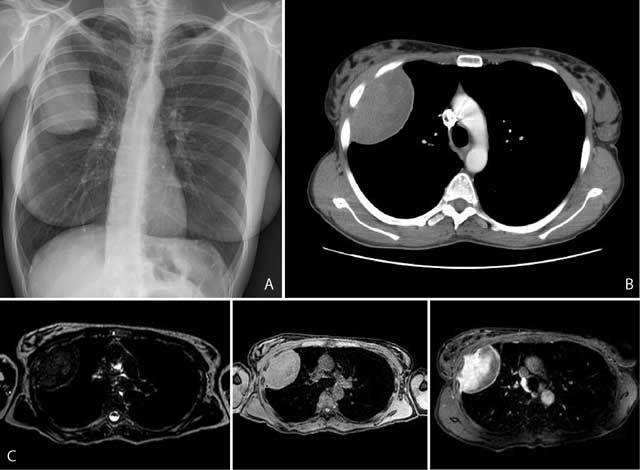


## Comment

Desmoid tumors, also known as aggressive fibromatosis, are benign soft tissue neoplasms. They are uncommon tumors accounting for less than 0.03% of all neoplasms. The overall incidence in the general population is 2–4 cases per million per year with a wide age range at diagnosis. Desmoid tumors have been described in numerous anatomic locations but are most prevalent in the abdomen or abdominal wall. The chest wall is the most common extra-abdominal location. True primary pleural desmoid tumors, like most probably in our patient, are exceedingly rare.

Compared to other desmoid tumors, intrapleural desmoids tend to remain asymptomatic for a long time and are larger at presentation. Unlike superficial chest wall tumors which will create a palpable mass, intrapleural tumors will not cause symptoms until they grow large enough to cause pain due to chest wall invasion or dyspnea due to compression of lung parenchyma.

The etiology of desmoid tumors is still not fully understood, but different factors, like previous trauma or surgical incision, hormonal influences and genetic association with familial adenomatous polyposis, are thought to play a role in their development and growth.

Imaging is necessary for surgical planning, delineation of the tumor, and postoperative follow-up. The radiologic appearance is not pathognomonic however and biopsy is mandatory for definite diagnosis. The imaging characteristics of intra-thoracic desmoids have been described on several imaging modalities and are similar to those of desmoids in other locations. CT and MRI are the predominantly used techniques. On CT they mostly appear as well-defined, isodense to slightly hypodense lesions with variable contrast enhancement. Masses with a higher collagen content can be hyperdense. CT is excellent for depicting bony involvement. On MRI most desmoids are homogeneously isointense on T1WI and heterogeneously, slightly hyperintense on T2WI or STIR images. After gadolinium moderate to strong inhomogeneous enhancement can be seen. These imaging appearances can vary depending on the proportions of cellular tissue, myxoid tissue and collagen in the tumor. Lesions with higher cellularity and lower collagenisation will have a higher signal intensity on T2WI and will demonstrate more intense contrast enhancement. Bands of hypo-intense signal on all sequences are frequently reported in these tumors and probably reflect islets of collagenous tissue. These bands are characteristic of fibromatosis but not specific for it.

The main differential on imaging is with a malignant soft tissue sarcoma (particularly fibrosarcoma and malignant fibrous histiocytoma). Desmoids show an infiltrative growth pattern, cross fascial bounderies and do not show central necrosis, even when large. Most soft tissue sarcomas are space-occupying intramuscular lesions that compress rather than infiltrate adjacent tissues. Contrary to desmoids, soft tissue sarcomas respect fascial bounderies and often have central necrosis. When solely considering true primary pleural desmoid tumors, the differential list is more extensive and also includes localized fibrous tumor of the pleura, inflammatory pseudotumor, fibroma, mesothelioma, desmoplastic fibroblastoma and metastatic disease.

Desmoid tumors have no metastatic potential but show strong local invasiveness and therefore the most appropriate therapy is broad, complete resection with negative margins. But even with aggressive surgery there is a high risk of recurrence, which is more frequent in extra-abdominal desmoids. If an operation is not possible or incomplete, additional multimodal therapies are needed and being researched.

## Competing Interests

The authors declare that they have no competing interests.
